# Improving the Classification Accuracy for Near-Infrared Spectroscopy of Chinese *Salvia miltiorrhiza* Using Local Variable Selection

**DOI:** 10.1155/2018/5237308

**Published:** 2018-01-29

**Authors:** Lianqing Zhu, Haitao Chang, Qun Zhou, Zhongyu Wang

**Affiliations:** ^1^Beijing Key Laboratory for Optoelectronic Measurement Technology, Beijing Information Science & Technology University, Beijing 100192, China; ^2^School of Instrumentation Science & Opto-Electronics Engineering, Beihang University, Beijing 100191, China; ^3^Department of Chemistry, Tsinghua University, Beijing 100084, China

## Abstract

In order to improve the classification accuracy of Chinese *Salvia miltiorrhiza* using near-infrared spectroscopy, a novel local variable selection strategy is thus proposed. Combining the strengths of the local algorithm and interval partial least squares, the spectra data have firstly been divided into several pairs of classes in sample direction and equidistant subintervals in variable direction. Then, a local classification model has been built, and the most proper spectral region has been selected based on the new evaluation criterion considering both classification error rate and best predictive ability under the leave-one-out cross validation scheme for each pair of classes. Finally, each observation can be assigned to belong to the class according to the statistical analysis of classification results of the local classification model built on selected variables. The performance of the proposed method was demonstrated through near-infrared spectra of cultivated or wild *Salvia miltiorrhiza*, which are collected from 8 geographical origins in 5 provinces of China. For comparison, soft independent modelling of class analogy and partial least squares discriminant analysis methods are, respectively, employed as the classification model. Experimental results showed that classification performance of the classification model with local variable selection was obvious better than that without variable selection.

## 1. Introduction

*Salvia miltiorrhiza* (*S. miltiorrhiza*), named “Danshen” in China, has been considered as an important component of traditional Chinese medicines (TCMs) [1]. The most important and frequent clinical application of *S. miltiorrhiza* is mainly employed for treatment of various cardiovascular diseases, including coronary artery disease, hyperlipidemia, hypertension, arrhythmias, and stroke by either alone or in combination with other herbal ingredients [2]. As is known, the efficacy and quality of *S. miltiorrhiza* can vary considerably according to the different origins with different climate, soil, and planting conditions [3, 4]. So, the discrimination of the geographical origins and planting conditions is a crucial issue. Unfortunately, in order to make storage and distribution easier, *S. miltiorrhiza* is typically preserved in the powder form. It is difficult to identify and discriminate the *S. miltiorrhiza* powder without expert training, which is becoming an obstacle to the modernization and internationalization of *S. miltiorrhiza* [5]. With the growing demand for *S. miltiorrhiza*, the discrimination of the cultivation area with easy, reliable, and rapid analytical approaches has been paid more and more attention.

Nowadays, NIR spectroscopy has proven its effectiveness for both qualitative and quantitative analyses because of the characteristics towards the analyzed sample such as fastest, low cost, and nondestructive [6–10]. Associated with pattern recognition techniques, it becomes a powerful tool for analysis of pharmaceutical mixtures [6, 11] and discrimination of geographical origin of herbal medicines [12, 13]. Here, the majority of pattern recognition methods are soft independent modelling of class analogy (SIMCA) [14–17] and partial least squares discriminant analysis (PLS-DA) [18–20].

Due to the complexity of herbal medicines, spectra suffer from a high grade of band overlapping. For this problem, variable selection was used in quantitative analyses [21–23]. Nevertheless, most of the variable selection methods applied in qualitative analyses are just a simple choice of waveband according to the spectral variation or absorption feature [4, 17, 24]. In this way, the selected variables are not proper for every observation, and the improvement of classification accuracy is not obvious.

In this paper, a novel local variable selection (L-VS) strategy was proposed for discrimination of Chinese *S. miltiorrhiza* according to its geographical origins and planting conditions. With this method, the multiclassification was transformed into several two-classification problems. Firstly, the most proper variables for each pair of classes were selected based on a new evaluation criterion considering both classification error rate and best predictive ability under the leave-one-out cross validation scheme. Then, applying the new observation into each local classification model which was built on selected variables, a series of class labels have been supplied. Finally, the classification decision of the new observation is identified by the statistical analysis of class labels. As comparison, the SIMCA and PLS-DA models combined with L-VS and SIMCA and PLS-DA based on the full spectrum were also established, respectively.

## 2. Materials and Methods

### 2.1. Sample Preparation

In this work, a total of 94 *S. miltiorrhiza* samples grown in wild or cultivated conditions were collected from 8 geographical origins in five provinces of China (Shandong, Henan, Sichuan, Hebei, and Yunnan) and used to perform the analysis. The origins, planting conditions, and numbers of all observations are summarized in Table 1. All samples were identified by experts from the China Institute of Traditional Chinese Medicine. Each sample of *S. miltiorrhiza* was dried and grounded into powder (200 meshes) before NIR measurement. It is worth pointing out that raw spectral data have been divided into 13 classes in the vertical direction (sample's direction) and 57 intervals in the horizontal direction (wavelength's direction).

### 2.2. NIR Spectra

NIR spectra of sample powders were acquired by a diffuse reflectance mode with a Spectrum One NTS Fourier-transform near-infrared spectrometer (PerkinElmer, USA). Spectra were recorded from 10000 cm^−1^ to 4000 cm^−1^ at 2 cm^−1^ resolution by coadding 32 scans using an integrating sphere. To reduce noise, each sample was measured 5 times, and the mean spectrum for each sample was used in the next analysis. Meanwhile, the temperature was kept around 25°C and at a steady humidity level in the laboratory.

### 2.3. Spectral Processing

All the computations were carried out in MATLAB software, version R2012b. The partial least squares (PLS) and principal component analysis (PCA) model calculations were performed with MATLAB functions that came from Stats' toolbox. And homemade routine was also employed for algorithms of SIMCA and PLS-DA. The raw spectra contain interference, such as high-frequency noise, baseline drift, background, and so on. Thus, before the classification stage, some preprocesses must be taken to weaken interference in spectra. The Savitzky–Golay (SG) method and the 2nd derivative spectra were adopted in this work. In the Savitzky–Golay smoothing process, polynomials of orders 1 and 21 on both sides were performed. And the wavelength interval used to calculate the 2nd derivative spectra was fixed at 40 cm^−1^.

**Figure 1:**
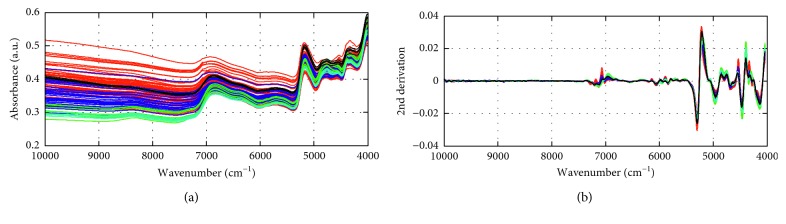
Raw near-infrared spectra (a) and the 2nd derivation preprocessed near-infrared spectra (b) of the *Salvia miltiorrhiza* observations.

The raw NIR spectra and the 2nd derivation preprocessed spectra of the 94 *S. miltiorrhiza* observations are shown in Figure 1. Figure 1 displays the raw NIR spectra of the *S. miltiorrhiza* observations in the region between 10000 and 4000 cm^−1^. From Figure 1, it can be appreciated that spectra of the *S. miltiorrhiza* observations show a very high similarity, and significant shifts were observed. However, by implementing the 2nd derivative, baseline drifts are eliminated and variations in the raw spectra are enhanced. The 2nd derivative spectra which are calculated after the SG smoothing are shown in Figure 1. As shown, it is still difficult to distinguish the spectral differences based on cultivation area and planting conditions, so the qualitative classification method is necessary.

### 2.4. Local Variable Selection Strategy

Classification method based on full-spectrum data range ignores directions in the variable space which are spanned by irrelevant, noisy variables. From a classification perspective, the large number of irrelevant variables may yield incorrect classification results. In order to minimize the influence of such irrelevant variables, variable selection is usually necessary.

In this paper, a novel L-VS strategy is proposed. According to the fundamental concept of local strategy, the spectral data have been divided into several pairs of classes, reducing the complexity by transforming the multiclassification into integration of several two-classification problems. Here, let *N*_1_ be the total number of classes and the calculation of the number of the class pair *N*_2_ be the mathematic combination problem, which is given by

(1) $$\begin{array}{c} N_{2}=C_{N_{1}}^{2}=\frac{\left(N_{1}!\right)}{\left(2!\left(N_{1}-2\right)!\right)}=\frac{N_{1}\left(N_{1}-1\right)}{2}. \end{array}$$

The main idea of the proposed L-VS is that, for each pair of classes, a specific set of variables has been selected from the full-spectrum data on the basis of performance of the local classification model between these two classes.

For the purpose of decreasing computation, the L-VS strategy also shares conceptual similarities with interval PLS (iPLS) [25], which develops local classification models on equidistant subintervals of the full-spectrum region, thereby focusing on the spectral region where information is optimally preserved. The critical issue that is important for the successful implementation of the L-VS is the size of the interval. Obviously, improper size of the interval destroys the model. Too many variables lose the effects of smaller peaks. On the contrary, too few variables may eliminate some useful redundancy and amplify the influence of each variable in the final model. In either case, the false classification will be caused. These two respects may be somewhat contradictory, so a reasonable compromise solution is applied in this paper, where the size of the subinterval is a relative big number and the resolution has a relative small number. For example, the spectral data of *S. miltiorrhiza* which has 3000 variables were divided into 57 intervals with a variable resolution of 50, and each interval will be made by 200 variables. This means that the first subinterval ranges between the 1st and 200th variables, the second subinterval between the 51st and 250th variables, and so on.

In addition, a novel evaluation criterion which incorporates both classification error rate and predictive ability of the classification model has been employed in the process of variable selection. And the optimized spectral region has been selected along the direction of the minimum incorrect classification rate and best predictive ability under the leave-one-out cross validation scheme for each pair of classes.

It should be noted that the proposed method is simply a “univariate” approach that does not consider combinations between spectral regions. Concomitant with the combination is a global optimization problem which corresponds to a much longer computation time even in small datasets. Therefore, it is proposed to select the optimal spectral region using the “univariate” approach in this paper. Subsequent experimental results have also proved that the use of the “univariate” approach makes L-VS more efficient and fast for large datasets, while obtaining an acceptable classification. In addition, different classification models have different ways to represent the predictive ability, and deciding which to choose is described in the following section.

### 2.5. Classification Decision

For each pair Class (*i*, *j*), the local classification model based on selected spectra is utilized to assign the new observation to Class_*i*_ or Class_*j*_. Traverse through all class pairs, the classification result of each new observation is a series of class labels. To make objective classification decisions using the class labels, there is a need to develop some decision rules for this purpose. Here, the classification decision is based upon the statistic occurrence frequency, and each observation can be assigned to belong to the class with the highest occurrence frequency of class labels.

### 2.6. SIMCA

SIMCA is one of the most commonly used class-modelling techniques for multivariate classification. In SIMCA classification, the residual of PCA modelling performed for each class is utilized to identify whether an unknown observation belongs to that class or not. During the step of local variable selection, the best classification performance for SIMCA is obtained when there are both lowest classification error rate and largest distance between classes.

(2) $$\begin{array}{c} \text{IC}=\frac{N_{\text{ic}}}{\left(N_{c}+N_{\text{ic}}\right)}, \end{array}$$

where IC is the classification error rate, *N*_*c*_ is the number of correct classifications, and *N*_ic_ is the number of incorrect classifications.

(3) $$\begin{array}{c} D_{ij}^{2}=\frac{\left(S_{ij}^{2}+S_{ji}^{2}\right)}{\left(S_{i}^{2}+S_{j}^{2}\right)}, \end{array}$$

where *D*_*ij*_^2^ is the distance between Class_*i*_ and Class_*j*_, *S*_*ij*_^2^ is the residual variance of the PCR model which is developed for fitting the Class_*j*_ and based on Class_*i*_, and *S*_*i*_^2^ is the residual variance of the PCR model based on Class_*i*_ with leave-one-out cross validation.

### 2.7. PLS-DA

In PLS-DA, all observations are assigned a class-specific numerical value *y* based on the geographical origins and planting conditions. For the two-class case, the *y* value should be as close to 1 as possible to belong to a certain class or close to −1 otherwise, and a simple 0 threshold has been employed to determine class belonging. The lowest classification error rate and root mean square error of prediction (RMSEP) in cross validation during the leave-one-out procedure are the criteria for guiding the variable selection to the optimum. Here, the classification error rate, as mentioned above, is calculated by (2), and RMSEP is given by

(4) $$\begin{array}{c} \text{RMSEP}=\sqrt{\sum_{j=1}^{N}\frac{{\left(y_{j}-{\hat{y}}_{\left(j\right)}\right)}^{2}}{N},} \end{array}$$

where *y*_*j*_ is the class-specific numerical value of the *j*th sample in the training set (union of two classes), ${\hat{y}}_{\left(j\right)}$ is the predicted value with PLSR in cross validation without the *j*th sample, and *N* is the total number of samples in the training set.

## 3. Results and Discussion

### 3.1. Data Exploration Using PCA

**Figure 2:**
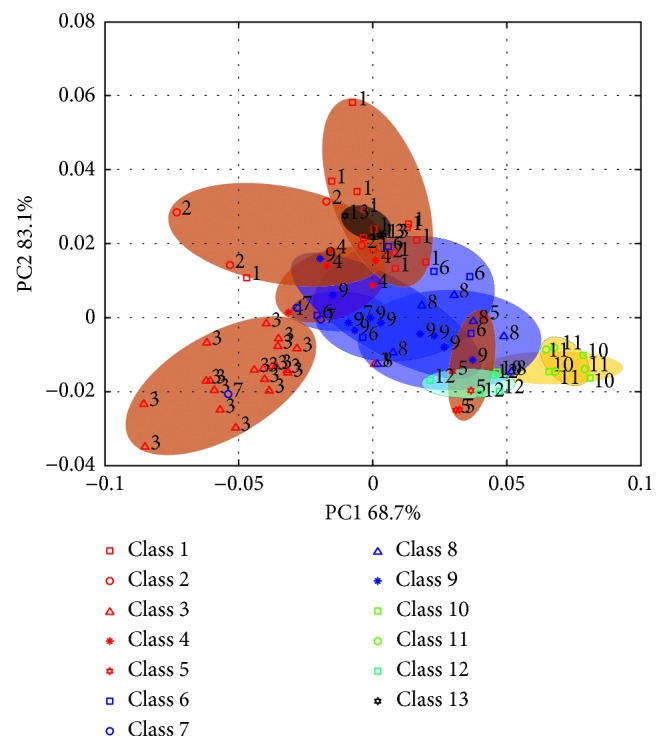
Score plots of the first principal component versus the second obtained from principal component analysis. PC1: the first principal component and PC2: the second principal component.

PCA was utilized initially to examine the qualitative difference of all the *S. miltiorrhiza* samples in the principal component (PC) space. As the explained variance of per principal component decreases with increasing number of PCs, the first two PCs encompass almost all variance, whereas the higher PCs are dominated by noise. In this paper, therefore, the PCA model was built from the total samples using 2 PCs explaining the variance in the data after applying the preprocessing described in the experimental section (S-G filtering, 2nd derivative). In Figure 2, two obtained score plots are depicted. It can be seen that the first PC (PC1) explained 68.7% and the second one (PC2) 14.4%. However, the *S. miltiorrhiza* samples (with different cultivation areas or planting conditions) usually have same compositional attributes, which are expressed as highly overlapped, and similar NIR absorption or reflectance. Due to this, the first two PCs, although representing the main part of the data variance, might not be powerful enough to achieve class separation. As shown in Figure 2, the distribution of PC1 and PC2 score plots was partly overlapped between different classes, especially Class 6, 7, 8, 11, and 13, which has no or only a slight clustering tendency. This is also proved by the results of the classification model based on entire spectra in Section 3.3. The correct identified rates of Class 6, 7, 8, and 11 are obviously lower than those of other classes. As can be seen, it is difficult to differentiate all the *S. miltiorrhiza* samples using the entire NIR spectra, particularly when the overlapping of PC1 and PC2 score plots for classes is observed, which motivates the use of the variable selection method.

### 3.2. Variable Selection

In order to improve the discrimination performance of the classification model, selecting the specific wave bands for each class is necessary. Firstly, the full-spectrum region was divided into 57 intervals with a variable resolution of 50, and each interval will be made by 200 variables. So, the *i*th interval related to the wavenumber from 10000 − 2 × (50 × *i* + 150) cm^−1^ to 10000 − 2 × (50 × *i* − 49) cm^−1^. Then, SIMCA and PLS-DA have been applied as the local classification model, respectively, to select the most proper region for each class pair.

#### 3.2.1. Local Variable Selection Based on SIMCA

**Figure 3:**
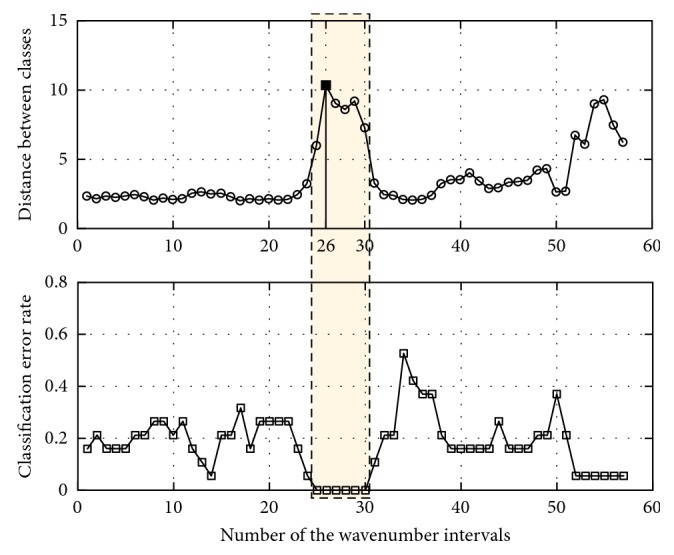
Variation of the classification performance of SIMCA with spectral intervals for Class 1 and 2. SIMCA: soft independent modelling of class analogy.

Here, the classification performance of SIMCA is characterized by the classification error rate and distance between classes. The optimal interval for each pair of classes, which provides the maximum distance between classes with the minimum classification error rate, has been selected for further study.  Figure 3 illustrates this concept using the Class 1 and 2 sample NIR data. In this plot, the classification error rate and distance between Class 1 and 2 are calculated by the SIMCA classification model with the spectra data in each interval. By using the principle of the minimum classification error rate, the 25th–30th intervals have been selected as the feasible region of the optimal interval. Furthermore, compared with the distances between Class 1 and 2 in the feasible region, the 26th interval with the maximum distance has been determined as the optimal interval for Class 1 and 2.

**Figure 4:**
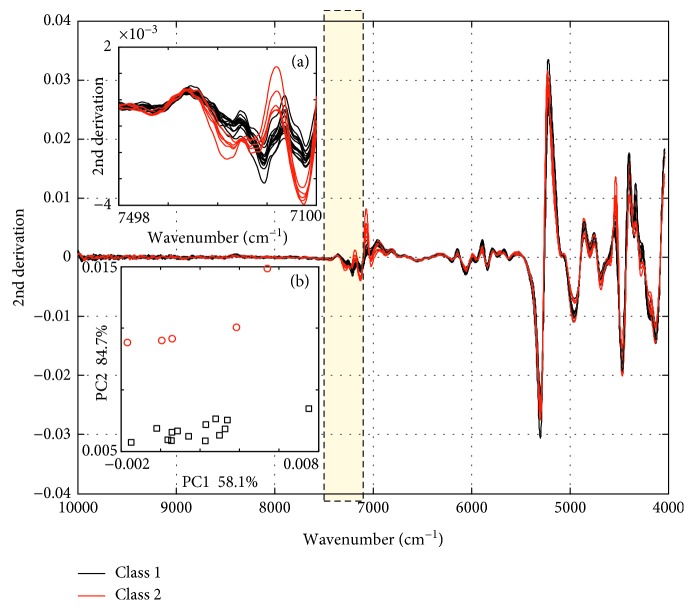
Comparison of Class 1 and 2 in the spectral space and principal component space. (a) The 2nd derivative preprocessed spectra of Class 1 and 2 between 7100 cm^−1^ and 7498 cm^−1^. (b) The first and second scores of Class 1 and 2 using the 2nd spectra between 7100 cm^−1^ and 7498 cm^−1^.

The comparison of Class 1 and 2 in the spectral space and principal component space after L-VS (based on SIMCA) is depicted in Figure 4. As shown in Figure 4, the spectral difference of Class 1 and 2 between 7100 cm^−1^ and 7498 cm^−1^ is more apparent as compared to that in the full spectrum. Further principal component analysis shows that the first two principal components together explained 84.7% of the variation, and clustering according to origins was clearly observed in Figure 4. As discussed, the result further indicates that the range from 7598 cm^−1^ to 7200 cm^−1^ is the most proper region which determines the cultivation area or planting conditions of the *S. miltiorrhiza* samples in Class 1 and 2.

**Figure 5:**
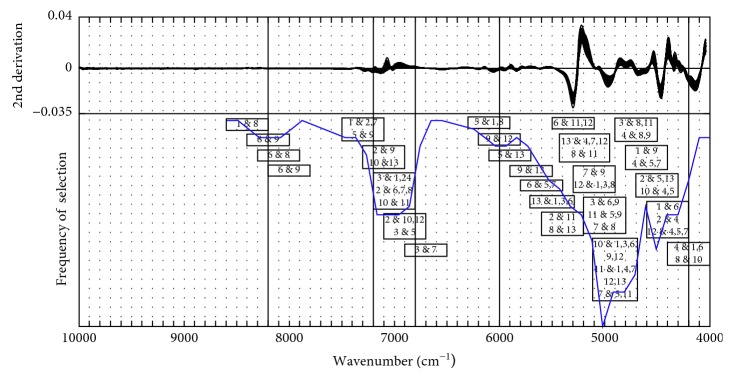
Location of the selected regions by L-VS based on the SIMCA classification method. L-VS: local variable selection; SIMCA: soft independent modelling of class analogy.

The 2nd derivative spectra of all the *S. miltiorrhiza* samples are presented in Figure 5. At the lower end, the curves show a great variety, while at the higher end, the curves vary less. And the distribution of the selected wavenumber for each pair of classes based on the SIMCA classification method and the fitting curve of the frequency of selection (blue solid line) are also shown in Figure 5. It can be seen that the optimal selected variables basically focus on the region with larger spectral differences which are mainly concentrated nearby 8200 cm^−1^, 7200∼6800 cm^−1^, and 6000∼4200 cm^−1^. It is worth to note that 1&2, 7 represents two pairs of classes: Class 1 and 2 and Class 1 and 7, and they have the same optimal variables between 7498 cm^−1^ and 7100 cm^−1^.

#### 3.2.2. Local Variable Selection Based on PLS-DA

**Figure 6:**
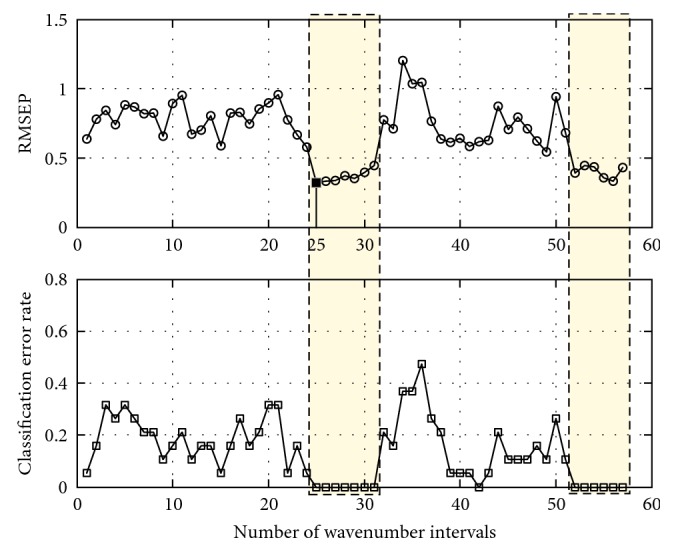
Variation of the classification performance of PLS-DA with spectral intervals for Class 1 and 2. RMSEP: root mean square error of prediction; PLS-DA: partial least squares discriminant analysis.

The same analysis was performed using PLS-DA. In this case, the PLS-DA was used as the classification model for local variable selection. In the L-VS process, the performance of PLS-DA built upon different regions was evaluated by both RMSEP and classification error rate via leave-one-out cross validation, the results of which are summarized in Figure 6. Based on the minimum classification error rate, two spectral regions have been selected: the first one is between the 25th and 31st intervals (7598∼6600 cm^−1^) and the second one includes the 52nd and 57th intervals (4898∼4038 cm^−1^). Furthermore, the RMSEP of the PLSR model between each pair of classes on different regions has been considered, and the 25th interval (7598∼6600 cm^−1^) was regarded as the most desirable region to generate the favorable classification performance.

**Figure 7:**
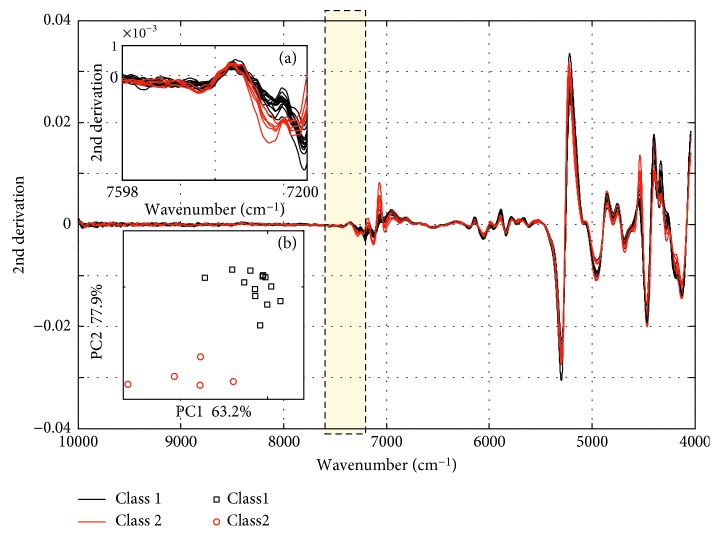
Comparison of Class 1 and 2 in the spectral space and principal component space. (a) The 2nd derivative preprocessed spectra of Class 1 and 2 between 7200 cm^−1^ and 7598 cm^−1^. (b) The first and second scores of Class 1 and 2 using the 2nd spectra between 7200 cm^−1^ and 7598 cm^−1^.

As shown in Figure 7, the difference of Class 1 and 2 between 7598 cm^−1^ and 7200 cm^−1^ is also more apparent as compared to that in full spectrum both in the spectral space and principal component space. This means that the first two principal components based on the range from 7598 to 7200 cm^−1^ explained high variance and contained sufficient information to achieve complete Class 1 and 2 separation. Therefore, we conclude that the narrow range from 7598 to 7200 cm^−1^ was best suited to discriminate the *S. miltiorrhiza* samples in Class 1 and 2, when the PLS-DA was applied as the classification method.

**Figure 8:**
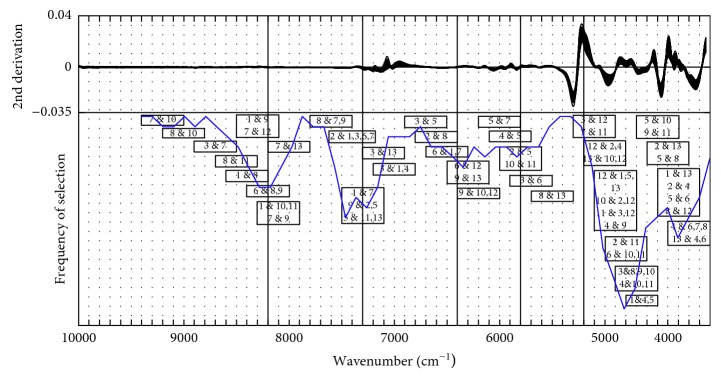
Location of the selected regions by L-VS based on the PLS-DA classification method. L-VS: local variable selection; PLS-DA: partial least squares discriminant analysis.

Figure 8 displays the distribution of the selected regions by L-VS based on the PLS-DA classification method. It is obvious that the regions with larger spectral differences are nearly selected as proper regions for class pairs. Additionally, along with the frequency of selection curve, some variables located in the region with lower spectral differences (such as 8500∼8000 cm^−1^) are also selected by L-VS.

Generally speaking, the selected regions with the L-VS method based on SIMCA and PLS-DA have some similarities. And spectral features contained in these selected regions and corresponding assignments are listed in Table 2. Basically, the main spectral contributions are derived from stretching vibrations of C–H, O–H, and N–H bonds. The region between 4400 and 4200 cm^−1^ is dominated by the combination bands of C–H. The region 4700∼4600 cm^−1^ is characteristic for the N–H stretching vibration and combination bands of C=O. The two bands at 5200∼5000 cm^−1^ and 7200∼7000 cm^−1^ correspond to the combination and the first overtone of the O–H stretching vibration. Near 8200 cm^−1^, the second overtone of C–H in CH_2_ can be found.

### 3.3. Comparative Performance of Various Methods

As comparison, the correct identified rates of all the *S. miltiorrhiza* observations obtained by SIMCA and PLS-DA with full spectrum and SIMCA and PLS-DA combined with L-VS strategy and traditional VS have been summarized in Table 3. Limited by the amount of the data, the classification procedure is similar to cross validation, in that one sample is left out from the training set and the remaining observations are used to discriminate the leftout observation.

It is generally believed that PLS-DA typically outperforms SIMCA in the correct classification rates, because the PLS-DA is a supervised method, which takes account of information of both spectra and class properties. So, the classification rates from PLS-DA based on full spectrum are generally larger than those from SIMCA in Table 3. However, there are still many class with lower correct identified rates, such as Class 6, 7, and 8, whose score plots are seriously overlapped. With the traditional variable selection, only the 7400 to 3000 cm^−1^ range was used since most sharp spectral features appeared to be located in this region. But, in fact, the performance of the classification model with these selected variables showed no improvement. Compared with full-spectrum and traditional variable selection, the L-VS has the remarkable improvement on the correct identified rates of both SIMCA and PLS-DA. Especially adopting PLS-DA combined with L-VS, all the *S. miltiorrhiza* observations are perfectly classified according to their geographical origins and planting conditions. Therefore, we conclude that the proposed L-VS strategy is a powerful tool for improving the classification accuracy of Chinese *Salvia miltiorrhiza*.

## 4. Conclusions

Throughout this paper, a local variable selection strategy has been proposed to improve the classification accuracy for discrimination of Chinese *S. miltiorrhiza*. And a novel evaluation criterion, considering both classification error rate and predictive ability of the classification model, has been employed in the process of variable selection. For comparison, SIMCA and PLS-DA have been used as the classification method, respectively. With the NIR spectral data of *S. miltiorrhiza* collected from 8 geographical origins in five provinces of China, it has been proved that the optimized variables containing sufficient clustering information can be selected by L-VS for each pair of classes. And the classification rates of the classification model combined with L-VS have been improved significantly. Especially, with the use of the PLS-DA classification model, all the *S. miltiorrhiza* observations were perfectly classified. In conclusion, the use of the near-infrared spectroscopy-integrated classification model combined with L-VS strategy can be developed into an accurate and high-performance method for discriminating Chinese *S. miltiorrhiza*.

## Figures and Tables

**Table 1:** Geographical origins and planting conditions of the *Salvia miltiorrhiza* observations.

Number of classes	Geographical origin	Planting condition	Number of samples
Class 1	Shandong, Li village	Wild (digging)	14
Class 2	Shandong, Shimen village	Cultivated for 1 year (digging)	Five
Class 3	Shandong, Shimen village	Cultivated for 2 years (digging)	21
Class 4	Shandong, Yishui	Wild (buy)	Five
Class 5	Shandong, Yishui	Cultivated (buy)	Five
Class 6	Henan, Lushi Mopanhe	Wild (digging)	Seven
Class 7	Henan, Lushi	Wild (buy)	Four
Class 8	Henan, Lingbao	Wild (digging)	Seven
Class 9	Henan, Lingbao	Cultivated (digging)	10
Class 10	Sichuan, Zhongjiang	Cultivated (buy, first level)	Four
Class 11	Sichuan, Zhongjiang	Cultivated (buy, second level)	Four
Class 12	Hebei, Anguo	Cultivated (digging)	Four
Class 13	Yunnan, Luxi	Wild (buy)	Four

**Table 2:** Position and assignment of the main absorption bands observed in the region between 10,000 and 4000 cm^−1^.

NIR band position (cm^−1^)	Remark (assignments)
4200∼4400	Combination bands of C–H
4600∼4700	Combination bands of C=O and N–H stretching
5000∼5200	Combination bands of O–H and N–H
5900∼6100	The first overtone of C–H
7200∼7000	The first overtone of O–H
8100∼8300	The second overtone of C–H in CH_2_

**Table 3:** Comparison of classification rates between different methods.

Set	Correct identified rate					
	Full spectrum		Traditional VS		L-VS	
	SIMCA	PLS-DA	SIMCA	PLS-DA	SIMCA	PLS-DA
Class 1	0.93	0.79	0.93	0.93	1.00	1.00
Class 2	0.80	0.60	0.80	0.80	1.00	1.00
Class 3	1.00	1.00	1.00	1.00	1.00	1.00
Class 4	0.40	1.00	0.40	1.00	1.00	1.00
Class 5	0.40	1.00	0.40	1.00	1.00	1.00
Class 6	0.71	0.86	0.57	0.71	1.00	1.00
Class 7	0.25	0.50	0.25	0.50	1.00	1.00
Class 8	0.57	0.71	0.57	0.86	1.00	1.00
Class 9	0.90	0.90	0.90	0.90	1.00	1.00
Class 10	0.75	1.00	0.75	1.00	1.00	1.00
Class 11	0.50	0.50	0.50	1.00	1.00	1.00
Class 12	0.75	1.00	0.75	1.00	1.00	1.00
Class 13	0.50	1.00	0.50	1.00	0.75	1.00

VS: variable selection; L-VS: local variable selection; SIMCA: soft independent modelling of class analogy; PLS-DA: partial least squares discriminant analysis.
